# The relationship between vitamin D and risk of atrial fibrillation: a dose-response analysis of observational studies

**DOI:** 10.1186/s12937-019-0485-8

**Published:** 2019-11-14

**Authors:** Xiao Liu, Wei Wang, Zhaochong Tan, Xin Zhu, Menglu Liu, Rong Wan, Kui Hong

**Affiliations:** 1 Cardiovascular Department, the Second Affiliated Hospital of Nanchang University, Nanchang, 330006 Jiangxi China; 2 Jiangxi Key Laboratory of Molecular Medicine, Nanchang, Jiangxi China

**Keywords:** Vitamin D, Atrial fibrillation, Dose-response, Meta-analysis. Cardiovascular disease

## Abstract

**Background:**

The relationship between serum vitamin D and atrial fibrillation (AF) or postoperative atrial fibrillation (POAF) in patients undergoing coronary artery bypass graft (CABG) is still debated. It is also unclear whether there is a dose-response relationship between circulating vitamin D and the risk of AF or POAF.

**Methods:**

The Cochrane Library, PubMed, and Embase databases were searched for relevant studies. We used a “one-stage approach” with a restricted cubic spline model to summarize the dose-specific relationships between serum vitamin D and AF. Relative risk (RR) was used to measure the effects in this meta-analysis.

**Results:**

In total, 13 studies were included with a total of 6519 cases of AF among 74,885 participants. Vitamin D deficiency (< 20 ng/ml) was associated with increased risks of AF (RR: 1.23, 95% CI: 1.05–1.43). In the dose-response analysis, the summary RR for a 10 ng/ml increased in vitamin D was 0.88 (95% CI: 0.78–0.98) and there was no evidence of a non-linear association, P_non-linearity_ = 0.86. In the age subgroup, high vitamin D (per 10 ng/ml increase) reduced the risk of AF in the older group (> 65 years) (RR = 0.68, 95% CI = 0.52–0.89) but not among young individuals (< 65 years) (RR = 0.87, 95% CI = 0.72–1.06). In addition, a strong association was found between a 10 ng/ml increased in vitamin D and POAF incident in the patient after CABG (RR: 0.44, 95% CI: 0.24–0.82).

**Conclusion:**

Our dose-response meta-analysis suggested serum vitamin D deficiency was associated with an increased risk of AF in the general population and POAF in patients after CABG. Further studies are needed to explore the age difference in the association between serum vitamin D level and the risk of AF and whether vitamin D supplements will prevent AF.

**Trial registration:**

This study has been registered with PROSPERO (International prospective register of systematic reviews)-registration number-CRD42019119258.

## Introduction

Historically, vitamin D is known for its important role in skeletal disease, [1–3]. The focus in recent decades has been on the risks of vitamin D and non-skeletal diseases, such as cardiovascular disease or atrial fibrillation (AF) [4]. AF is the most common cardiac arrhythmia in clinical practice and is associated with increased risk of morbidity. Vitamin D deficiency is common in many countries [5], only 23% of people would reach serum vitamin D concentration above 30 ng/ml [6]. A cause-and-effect relation between low vitamin D status and AF incident would be of considerable benefit to public health. However, unlike for skeletal disease, the evidence for serum vitamin D deficiency (< 20 ng/ml) and the risk of AF has been inconclusive [7, 8]. To date, results from several observational studies have suggested that patients with vitamin D deficiency were approximately twice as likely to have AF than patients with normal levels (> 30 ng/ml) [8–11]. Conversely, several prospective studies did not find this association [7, 12–14]. Thereafter, several articles have reviewed published studies and yielded conflicting results [7, 15, 16]. Alonso et al. did not find a clinically relevant association of circulating vitamin D per 1 standard deviation (8.5 ng/ml) decreased with AF risk [7]. In contrast, a meta-analysis concluded a weak but positive association between vitamin D deficiency and AF [15]. However, there are several limitations in the previous meta-analyses. For example, vitamin D levels were analyzed as either a categorical or continuous variable in the individual studies, so they could not pool all of the studies together. In addition, the shape of the dose-response association between vitamin D and AF had been explored. Moreover, several new research articles reported higher serum 25(OH) D is associated with new-onset AF after coronary artery bypass grafting (CABG) surgery [17–20]. Therefore, we performed a comprehensive meta-analysis to evaluate the shape of the dose-response relation between circulating 25(OH) D concentration and the risk of AF and post-operation AF (POAF) after CABG.

## Methods

This work has been performed according to PRISMA guidelines (http://www.prisma-statement.org; Additional file 1: Table S1) [21]. We systematically searched the PubMed, Embase databases and Cochrane Library up to March 10, 2019. Additional file 1: Table S2 provides a detailed description of the search strategy. Two researchers independently worked in the whole process of this meta-analysis from the literature search and selection to data analysis. Both randomized controlled trials and observational studies, reporting data about serum vitamin D level and AF were considered eligible for this meta-analysis. All discrepancies were resolved through discussion by the two authors. We used the robust error meta-regression method (REMR) for the dose-response analysis of the vitamin D level and AF [22, 23]. All statistical analyses were done by using Review Manager (RevMan) version 5.3 (The Cochrane Collaboration 2014; Nordic Cochrane Center Copenhagen, Denmark) and Stata software (Version 14.0, Stata Corp LP, College Station, Texas, USA). We used the Newcastle-Ottawa quality assessment scale (NOS) to evaluate the quality for all included studies [24], a NOS score of ≥6 stars was regarded as high-quality, otherwise, as low-quality studies [25, 26]. Full details of the literature search strategy, study selection criteria, quality assessment, and statistical analysis have been reported in the Supplement Methods (Additional file 1). This study has been registered with PROSPERO (International prospective register of systematic reviews)-registration number-CRD42019119258.

## Results

### Study selection

**Fig. 1:**
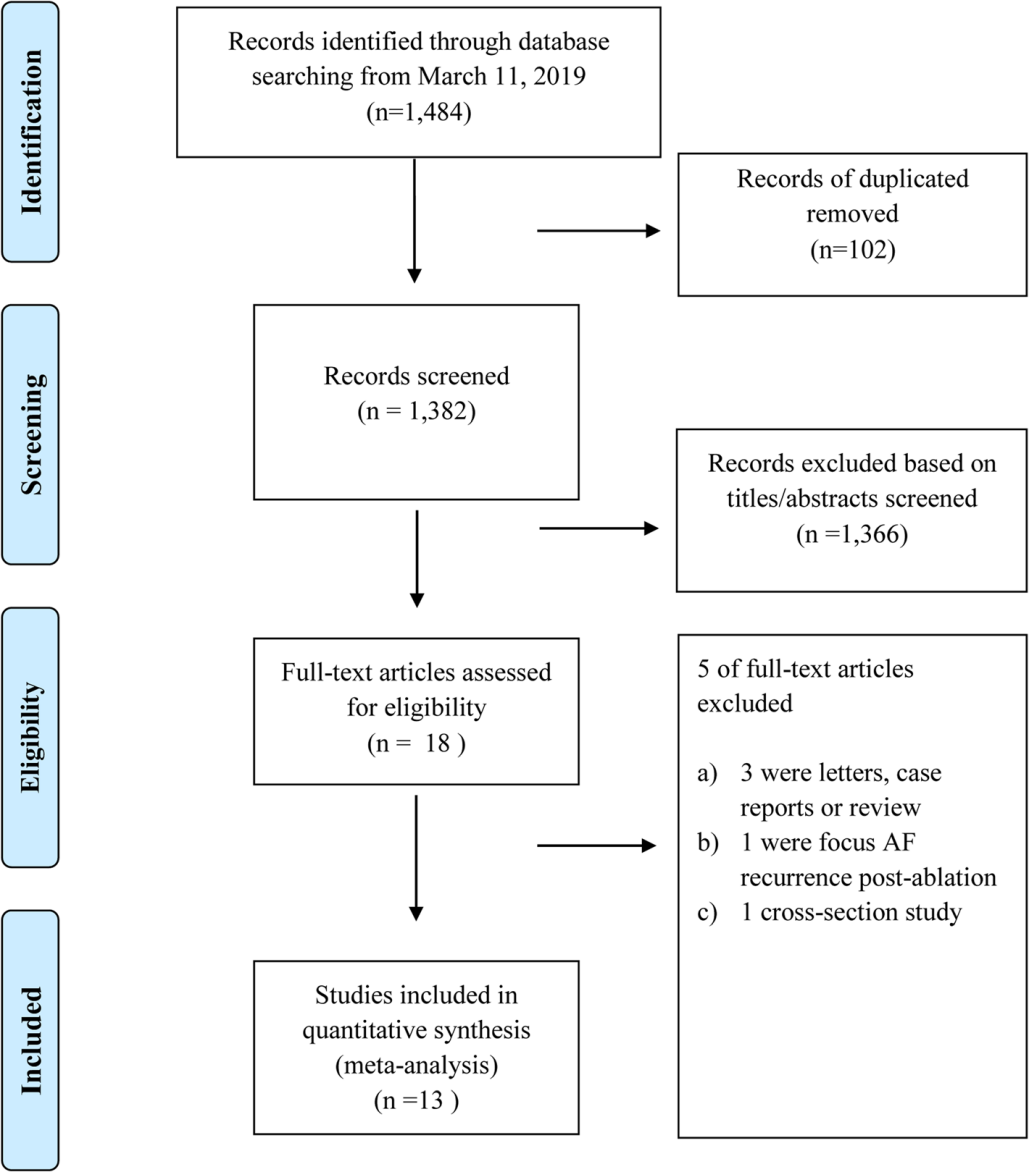
Flowchart of study selection

We identified 1484 studies in our initial database search. After removing duplicates and studies with inadequate information on vitamin D and AF, 20 studies were reviewed in more detail. Of these 20 studies, 5 were excluded for the following reasons: a) they were focused on recurrence AF (*n* = 1) [27]; b) they were reviews or case reports (*n* = 2) [15, 28]; or c) cross-section study (*n* = 1, 20]. Finally, 13 studies (14 reports) were included in this meta-analysis (Fig. 1).

### Study characteristics and quality

Detailed characteristics of the included studies are presented in Table 1. Thirteen studies (14 reports) with 6519 AF cases and 74,885 participants were included in this meta-analysis [7–14, 16–19, 29]. Overall, these studies were published between 2011 and 2018. The sample sizes of the included studies varied from 48 to 47,062. The mean age ranged from 57 to 77 years. The duration of follow-up across the studies varied from 48 h to 18 years. Among the 13 articles, six were cohort studies [7, 12–14, 16], four were nest-case-control (prospective case-control) studies [17–19, 29], four [8–11] were case-control studies. Nine studies [7–14, 16] examine the serum vitamin D status and AF and 4 [17–19, 29] focused POAF in patients undergoing CABG. Only two studies [10, 16] were scored as low quality with a NOS of 5 stars. The rest eleven [7–9, 11–14, 17–19, 29] were high quality (≥6 stars) (Table 2).

**Table 1:** Basic characteristics of the 13 articles included in the meta-analysis

Author, publication year, country	Study design, Follow up	Source of parcipant	Conducted season	Cases/N	Definition of AF, measurement of vitamin D	Mean age (years), male (%)	Expose level	RR (95%CI)	Adjustment for confounders
Rienstra, 2011, USA [12]	Prospective cohort, 9.9 years	Framingham Heart Study	NA	425/2930	ECG, competitive protein-binding assay and radioimmunoassay	65, 44	Continuous variable	0.99 (0.89–1.10)	Age, sex, BMI, PR interval, and cardiac murmur.
Chen, 2014, China [9]	Case-control, NA	Chinese PLA General Hospital	Winter	162/322	ECG, chemilumiscence assay	65, 45	< 20 ng/ml 21–29 ng/ml ≥ 30 ng/ml continuous variable	1.97 (1.31–2.97) 1.32 (1.06–1.66) ref. 0.4 (0.30–0.80)	Age, systolic blood pressure, hsCRP, LAD, LV end diastolic diameter, LVEF, and PASP.
Demir, 2014, Turkey [10]	Case-control, NA	Bursa Education and Research Hospital	Winter	198/298	NA, BioSource 25-OH Vit.D3-Ria-CT Kit	62, 40	Continuous variable	0.86 (0.786–0.94)	Medications, age, gender, and BMI.
Mathew, 2014, USA [14]	Prospective cohort, 7.7 years	MESA Study	NA	291/6398	ECG, hospital discharge diagnoses, inpatient and outpatient physician claims data	62, 53.5	Per 10 ng/mL	0.92 (0.81–1.03)	Age, gender, race/ethnicity, study site, attained education, low density, cholesterol, use of lipid-lowering medications, current smoking, diabetes, physical, activity, height, height squared, weight, urine albumin-creatinine-ratio, eGFR, systolic blood pressure, and use of hypertension medication.
	Prospective cohort, 8.0 years	CHS Study	NA	229/1350		77, 71.3	Per 10 ng/ml	1.00 (0.88–1.14)	
Ozcan,2015, Turkey [11]	Prospective case-control, NA	Ankara University Hospital	Winter and Spring	90/227	ECG, chemiluminescent immunoassay	68, 58	< 20 ng/ml ≥ 20 ng/ml Continuous variable	1.68 (1.18–2.64) Ref 0.86 (0.66–1.05)	Age, gender, BMI, smoking status, hyperlipidemia, medications, serum levels of creatinine, calcium, LAD, LAEF, and PASP
Vitezova, 2015, Netherlands [13]	Prospective cohort, 12 years	The Rotterdam Study	NA	263/3295	ECG, electrochemiluminescence immunoassay	71, 41	< 50 nmol/l 50–74 nmol/l ≥ 75 nmol/l	ref 0.82 (0.60–1.11) 0.76 (0.52–1.12)	Age, gender, income, education, BMI, physical activity, diet quality score, smoking status and season and year when the blood was drawn.
Emren, 2016, Turkey [17]	Prospective case-control study, NA	Residents in Afyonkarahisar	Winter and Spring	71/212	ECG, direct chemiluminescence immuno assay	63, 75	< 11.5 ng/ml ≥ 11.5 ng/ml	ref 0.95 (0.91–0.99)	Age, male sex, chronic HF, AF episodes, COPD, chronic renal failure, DM, rheumatic heart disease, metabolic syndrome, obesity, and inadequate use of beta blockers or RAS blockers.
Alonso, 2016, USA [7]	Prospective cohort, 18 years	Communities Study	NA	1866/12,303	ECG and Hospital discharge codes, high-sensitivity mass spectrom eter	57, 43	< 20 ng/ml 21–29 ng/ml ≥ 30 ng/ml	1.10 (0.96–1.26) 1.09 (0.97–1.22) ref	Age, sex, race, study centre, education, alcohol consumption, height, BMI, smoking status, physical activity, systolic and diastolic BPs, medication, DM, coronary heart disease, HF, hsCRP, NT-pro-BNP, and eGFR.
Belen, 2016, Turkey [8]	Case-control, NA	Hospital in Okmeydan	NA	96/180	ECG, high-performance liquid chroma tography	66, 54	Continuous variable	0.854 (0.805–0.907)	Age, gender, BMI, etiology and chronic HF stage.
Gode, 2016, Turkey [19]	Prospective case-control study, 5 day	Hospital in Istanbul	Winter	15/90	ECG, analysed in biochemistry laboratory	58, 78	≤ 30 ng/mL > 30 ng/mL	ref 0.856 (0.751–0.976)	LAD, creatinine, cholesterol and DM.
Skuladottir, 2016, Iceland [29]	Prospective case-control study, 72 h	The National University Hospital of Iceland	NA	66/118	Holter monitoring, MS/MS Vitamin D Kit	NA, 80	< 47.1 nmol/l ≥ 47.1 nmol/l	1.31 (0.54–3.16) ref	Age, BMI, smoking, peak postoperative C-reactive protein, preoperative plasma DHA level and valvular surgery or complex surgical procedure
Turin, 2018, USA [16]	Retrospective cohort, NA	Loyola University Medical Center	All seasons	2697/47,062	ICD-9 code data, liquid chromatography-MS	NA, 28	< 20 ng/ml ≥ 20 ng/ml	1.08 (0.95–1.22) ref	ACEI/ARB use
Özsin, 2018, Turkey [18]	Prospective randomized clinical, NA	Hospital in Bursa	Winter	50/100	ECG, Architect 25-OH vitamin D- Reagent Kit	60, 70	< 7.65 ng/dl ≥ 7.65 ng/dl	ref 0.855 (0.780–0.938)	Age, gender, history of hypertension, DM, preoperative drug use, EF, LAD, BMI, body surface area, aortic cross clamp time, cardiopulmonary bypass time.

*Abbreviations*: *AF* Atrial fibrillation, *HF* Heart failure, *hsCRP* high-sensitivity C-reactive protein, *LAD* Left atrium diameter, *LAVI* Left atrial volume index, *LV* Left ventricle, *LVEF* Left ventricular ejection fraction, *PASP* Pulmonary artery systolic pressure, *BMI* Body mass index, *BP* Blood pressure, *DM* Diabetes mellitus, *NT-proBNP* N-terminal of the prohorme B-type natriuretic peptide, *eGFR* estimated glomerular filtration rate, *HDL-C* High-density lipoprotein cholesterol, *LDL-C* Low-density lipoprotein cholesterol, *COPD* Chronic obstructive pulmonary disease, *RAS* Renin-angiotensin system, *POAF* Postoperative atrial fibrillation, *ECG,* Electrocardiography, *MESA,* Multi-Ethnic Study of Atherosclerosis, *CHS* Cardiovascular Health Study, *PLA* People’s Liberation Army, *ICD* International classification of diseases, *ACEI* Angiotensin-Converting Enzyme Inhibitors, *ARB* Angiotensin receptor blocker, *MS/MS* Cascade mass spectrometry, *DHA* Docosahexaenoic acid

**Table 2:** Quality assessment of cohort and case-control studies

Author (Publication Year)	Newcastle-Ottawa Scale									
	Selection			Comparability			Outcome			Total
	a	b	c	d	e	f	g	h	i	
Alonso, 2016 [7]	*	*	*	*	*	*	*	*		8
Belen, 2016 [8]	*			*	*	*	*	*		6
Chen, 2013 [9]	*			*	*	*	*	*		6
Demir, 2012 [10]				*	*	*	*	*		5
Ozcan, 2015 [11]	*			*	*	*	*	*		6
Rienstra, 2011 [12]	*	*	*	*	*	*	*	*		8
Vitezova, 2015 [13]	*	*	*	*	*	*	*	*		8
Mathew, 2014 (MESA) [14]	*	*	*	*	*	*	*	*		8
Mathew, 2014 (CHS) [14]	*	*	*	*	*	*	*	*		8
Turin, 2018 [16]	*	*	*	*			*			5
Emren, 2016 [17]	*		*	*	*	*	*	*		8
Özsin, 2017 [18]	*		*	*	*	*	*	*		8
Gode, 2016 [19]	*			*	*	*	*	*		6
Skuladottir, 2016 [29]			*	*	*	*	*		*	6

*MESA* Multi-Ethnic Study of Atherosclerosis, *CHS* Cardiovascular Health

### Categorical analysis of serum vitamin D on AF

**Fig. 2:**
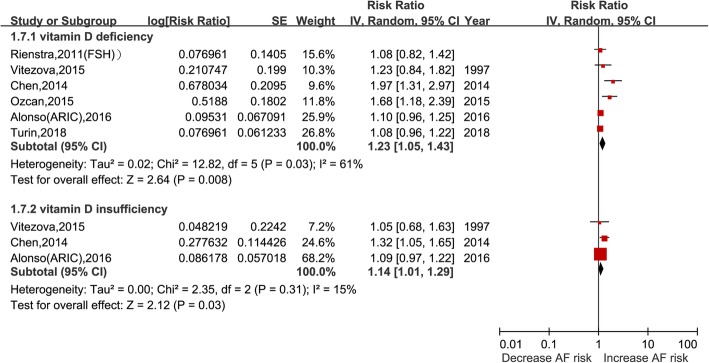
Vitamin D deficiency or insufficiency and risk of atrial fibrillation

Six studies with 5503 cases/66,139 participants were included [7, 9, 12, 13, 16, 18]. As shown in Fig. 2, vitamin D deficiency (< 20 ng/ml) was associated with increased risks of AF (RR: 1.23, 95% CI: 1.05–1.43; I^2^ = 61%; *P* = 0.008). The results were consistent both in the cohort (RR: 1.09, 95% CI: 1.01–1.19; I^2^ = 0%; *P* = 0.03) and case-control studies (RR: 1.80, 95% CI: 1.38–2.35; I^2^ = 0%, *P* < 0.001).

Three [7, 9, 13] studies with 2291 cases and 15,920 individuals assessed the association between 25(OH) D insufficiency (21–29 ng/ml) and risk of AF. The pooled RR suggested that vitamin D insufficiency is associated with the occurrence of AF (RR: 1.14, 95% CI: 1.01–1.29; I^2^ = 15%, *P* = 0.03) with no evidence heterogeneity.

### Dose-response association between circulating vitamin D and incident AF

**Fig. 3:**
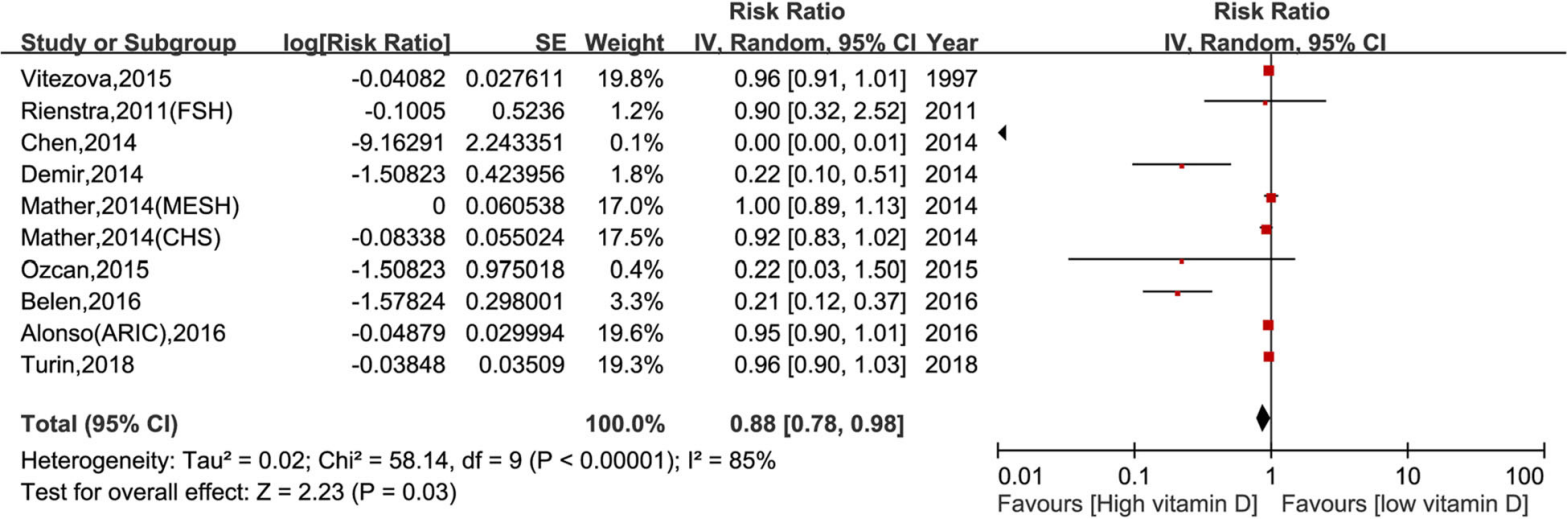
Vitamin D and risk of atrial fibrillation, per 10 ng/ml increase

Nine studies (10 reports) [7–14, 16] with 6364 cases/42,776 participants were included in the dose-response analysis of vitamin D and AF. The summary RR for a 10-unit increased of vitamin D was 0.88 (95% CI: 0.78–0.98, I^2^ = 85%, P = 0.03) (Fig. 3) and there was no evidence of a non-linear association, P_non-linearity_ = 0.34. To address the main source of heterogeneity, we implemented subgroup analyses according to study design, and significant evidence of heterogeneity was shown between study design subgroups (P_heterogeneity_ < 0.001). The results were similar in both cohort and case-control studies (Table 3). Moreover, high vitamin D (per 10 ng/ml increase) reduced the risk of AF in the older group (≥65 years) (RR = 0.68, 95%CI = 0.52–0.89, *P* = 0.005) but not among young individuals (< 65 years) (RR = 0.87, 95%CI = 0.72–1.06, *P* = 0.17) although no significant heterogeneity was found in the age subgroup (*P* = 0.15) (Table 3).

**Table 3:** Subgroup and sensitivity analysis- vitamin D and atrial fibrillation incident, per 10 ng/ml increase

Items		Number of studies	RR (95%CI)	*P*	P^*^_h (%)_	*P*^#^
Result of primary analysis		10	0.88 (0.78–0.98)	0.03	85	–
Mean age	< 65 years	3	0.87 (0.72–1.06)	0.17	83	0.15
	> 65 years	7	0.68 (0.52–0.89)	0.005	95	–
Study design	Case-control	4	0.13 (0.04–0.44)	< 0.001	74	0.001
	Cohort	6	0.96 (0.93–0.99)	0.007	0	–
Sample size	< 2000	5	0.21 (0.06–0.69)	< 0.001	96	0.01
	≥2000	5	0.95 (0.92–0.99)	0.005	0	–
Case	< 200	4	0.21 (0.06–0.69)	0.007	74	0.001
	≥200	6	0.97 (0.96–0.98)	0.001	0	–
Region	European	1	0.96 (0.91–1.01)	0.14	–	0.006
	America	5	0.96 (0.92–0.99)	0.02	0	–
	Asia	4	0.13 (0.04–0.44)	0.001	74	–
Study quality	< 6	2	0.49 (0.12–2.06)	0.33	92	0.32
	≥6	8	0.74 (0.61–0.91)	0.004	94	–
Repeated with fixed model		10	0.97 (0.96–0.98)	< 0.001	93	–
Exclusion of subjects						–
	Case-control omitted	6	0.96 (0.93–0.99)	0.007	0	–
	Low quality excluded	8	0.74 (0.61–0.91)	0.004	94	–
	Not adjusted age excluded	9	0.75 (0.64–0.88)	< 0.001	94	–
	Not adjusted BMI excluded	8	0.77 (0.66–0.89)	< 0.001	94	–
	Not adjusted sex excluded	9	0.75 (0.64–0.88)	< 0.001	94	–
	Not adjusted BMI excluded	6	0.74 (0.60–0.92)	88	0.006	–

^*^P value of heterogeneity. ^#^*P* for subgroup of subgroup. *BMI* body mass index; *RR* Ratio risk, *CI* confidence intervals

**Fig. 4:**
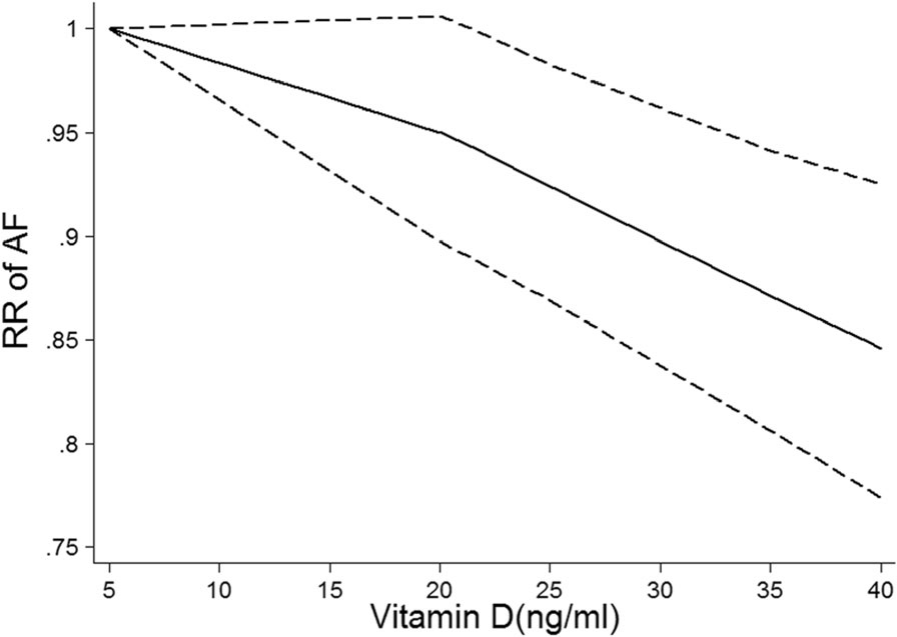
Vitamin D and risk of atrial fibrillation, nonlinear dose-response analysis. The solid line and the dashed lines represent the estimated relative risk and the 95% confidence interval, respectively

Then, we conducted a non-linear dose-response by using restricted cubic model and found an inverse relationship between vitamin D and AF (Fig. 4).

In subgroup and sensitivity analysis, the positive association between vitamin D and risk AF persisted in almost all subgroup analyses defined by the duration of follow-up, geographic location, number of cases, study quality and adjustment for confounding and potential intermediate factors (Table 3).

**Fig. 5:**
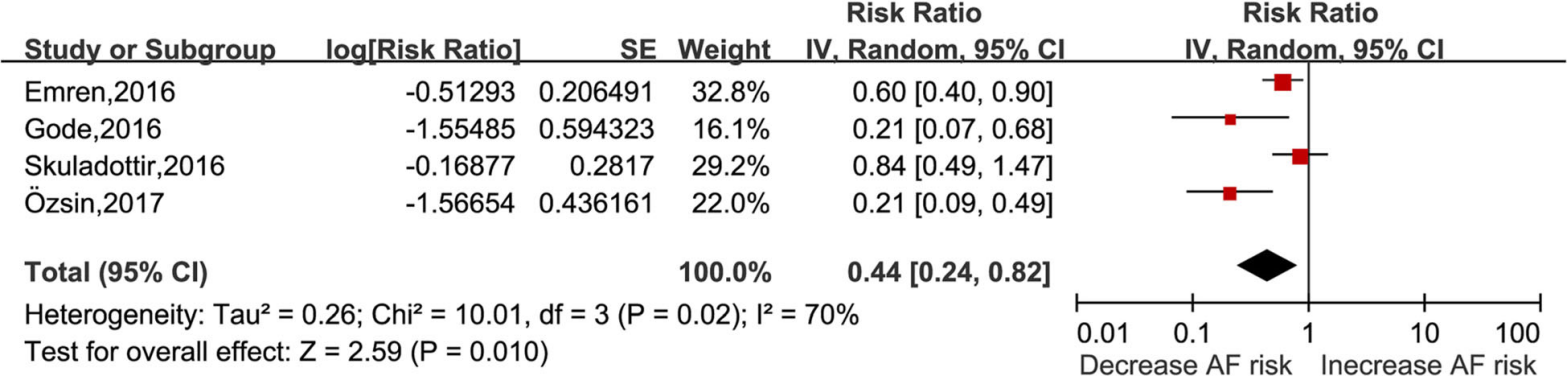
Vitamin D and risk of atrial fibrillation in patients undergoing CABG, per 10 ng/ml increase. CABG: Coronary artery bypass graft

Four studies [17–19, 29] with included 202 cases/520 patients were included in this analysis of vitamin D status and AF post-CABG. Serum vitamin D per 10 ng/ml increase were associated with decreased POAF incident (RR: 0.44, 95% CI: 0.24–0.82, I^2^ = 70%, *P* = 0.01) with modest heterogeneity (Fig. 5). The nonlinear dose-response analysis was not available because of limited information.

### Publication bias

There was some indication of publication bias with Egger’s test, *p* = 0.07, or with Begg’s test, *p* = 0.03 or by inspection of the funnel plot (Additional file 1: Figure S1-S3). Thus, we used the “trim-and-fill” method for the adjustment of publication bias. However, the results showed “no trimming performed and data unchanged”, which demonstrated that our results were stable. The publication bias of POAF was not conducted as limited studies (*N* < 10) according to the guideline [30].

## Discussion

To the best of our knowledge, this is the first meta-analysis to evaluate the dose-response association between vitamin D and AF. Our results by dose-response analysis suggested that vitamin D deficiency is a moderate predictor of AF. We found vitamin D deficiency (< 20 ng/ml) or vitamin inadequate (< 30 ng/ml) increased the risk of AF by 23% or 14%, respectively. Interestingly, the present dose-response analysis first showed vitamin D deficiency is also a moderate predictor for POAF, and novelty showed a 12% (AF) or 56% (AF post-CABG) increase in the RR per 10-unit increase in vitamin D with evidence of linear association.

Formerly, the role of circulating vitamin D in AF remains unclear. A meta-analysis found a vitamin D deficiency is associated with a 31% increased risk for AF [15]. However, another study which only pooled cohort studies did not support this association [7]. Our results showed there is an inverse association between vitamin D and risk of AF, both in cohort and case-control studies. However, significant heterogeneity between the subgroup of study design (cohort and case-control study) was observed. The association in cohorts was significantly weaker than that in case-control studies. Besides the recall bias, the heterogeneity in the study population appeared to be one of the potential reasons. The cohort studies were general population-based and the case-control studies mainly focused on patients with cardiovascular disease (CVDs). Recent studies also have shown that vitamin D was a potential connection with CVDs (eg. ischemic heart disease) and diabetes [15, 31]. Therefore, it might be reasonable to speculate that the association between vitamin D deficiency and AF may be amplified in patients with CVDs or at high risk of developing CVDs. However, considering the limited sample size, the role of vitamin D in patients with CVDs or at high CVDs risk need to be further studied.

The association between vitamin D deficiency and AF has several potential pathophysiological mechanisms. Inflammation has a crucial role in the pathogenesis of AF [32]. For example, C-reactive protein (CRP), the most robust and reproducible marker of vascular inflammation, could increase the risk of AF by up to two-fold [33]. Noticeable, low vitamin D status could directly or indirectly increase the synthesis of CRP [34]. Another important mechanism might be the activation of the renin-angiotensin-aldosterone system (RAAS). RASS plays an important role in both structural and electrical remodeling of the atrium. Studies in experimental animals showed vitamin D could inhibit the RAAS system [35]. Clinical researches showed that the use of ACEIs was associated with less atrial fibrosis, and the blockade of angiotensin II has been shown to have beneficial effects on electrical remodeling in human atrial tissue [36, 37]. Moreover, a previous meta-analysis also reported that inhibition of RASS might reduce the risk of developing new-onset atrial fibrillation [38]. Therefore, low vitamin D level might increase the AF risk secondary to its negative regulatory property of the RAAS.

It is not surprised that we found vitamin D deficiency is associated with increased POAF. Previous studies have suggested that deficiency of total vitamin D is associated with increased prevalence of electrocardiographic abnormalities (e.g. prolonging the duration of action potentials) [39]. Moreover, in a recent prospective cohort study of patients undergoing cardiac surgery, low total 25(OH) D levels were independently associated with the risk of major cardiac and cerebrovascular events [39]. Another study also showed an inverse relationship between serum 25(OH) D level and left atrial or AF recurrence in patients after undergoing catheter ablation [40]. However, considering the small sample size and short-term follow-up, the relationship between vitamin D and POAF need to be further confirmed in larger, well-designed studies.

We also studied the role of age in the present meta-analysis, we found low 25(OH) D level increased the risk of AF in the older individuals (age ≥ 65 years) but not young group (< 65 years). This result should be with caution in elder individuals. However, there was significant heterogeneity in the results, which might come from the study population or study design. When we excluded the case-control studies, both the older (RR: 0.96, 95%CI: 0.93–1.00) and young people (RR:0.94, 95%CI: 0.89–1.00) showed a weak association between vitamin D deficiency and risk of AF. Of note, these results were incosistent with the recent analysis of ARIC study [7], which showed that low vitamin D was a stronger indicator of AF in the youngest group (< 54 years) but not in the oldest (> 60 years), with an intermediate association in those aged 54–59 years. Therefore, based on current evidence, the age difference in the relationship between vitamin D and risk of AF is still unclear. Further prospective cohort studies are needed to clarify the age difference.

### Study limitations

The present meta-analysis has several limitations. First, this was a meta-analysis of observational studies, which cannot chiefly prove causation, and the unmeasured and insufficiently measured variables (e.g. seasonal variation in vitamin D) would result in the possibility of residual confoundings. However, most of our studies were performed during the winter or spring months, which could reduce this confounding factor. Second, due to data restriction, the impact of vitamin D supplements on AF was not analyzed and need to be further investigated as we previously discussed. Third, some studies suggested that available vitamin D may be a more reliable marker of vitamin D status than total 25 (OH) D. However, none of the included studies measured available vitamin D.

## Conclusion

Our dose-response suggested serum vitamin D deficiency was associated with an increased risk of AF in the general population and POAF in patients after CABG. Further studies are needed to explore if there is an age difference in the association between serum vitamin D level and the risk of AF and whether vitamin D supplements will prevent AF.

## Supplementary information

**Additional file 1.** Online Data Supplement.

## Data Availability

All data generated or analyzed during this study are included in this published article [and its supplementary information files].

## Abbreviations

AF: Atrial fibrillation
CABG: Coronary artery bypass graft
CI: Confidence interval
NOS: Newcastle-Ottawa Scale score
POAF: Postoperative atrial fibrillation
RR: Relative risk
