# Supplementary material for: The relationship between vitamin D and risk of atrial fibrillation: a dose-response analysis of observational studies
Source: Nutr J. 2019 Nov 14;18:73. doi: 10.1186/s12937-019-0485-8 (PMC6857145; doi:10.1186/s12937-019-0485-8)
Supplement: Supplementary file 1 — Additional file 1. Online Data Supplement. [file 12937_2019_485_MOESM1_ESM.docx]

**ONLINE DATA SUPPLEMENTAL**

**The Relationship between Vitamin D and Risk of Atrial Fibrillation: A Dose-response Analysis of Observational Studies**

**Running title:** Vitamin D and Atrial Fibrillation

Xiao Liu^a^, MD*; Wei Wan^a^, MD*, M.D., Zhaochong Tan^a^, M.D., Xin Zhu^a^, M.D., Menglu Liu^a^, M.D, Rong Wan^a^ Ph.D., Kui Hong^a,b^, MD, PhD

**SUPPLEMENTAL METHODS**

**Literature Search**

We systematically searched the Cochrane Library, PubMed, and Embase databases for eligible studies before March 10, 2019.Two groups of keywords (linked to vitamin D, and AF, respectively) were combined using the Boolean operator "and". Table S1 provides a detailed description of the search strategy in the aforementioned electronic databases. In addition, we searched the reference lists of two review[1, 2] or other relevant publications to identify further studies. No language restrictions were applied in the whole literature search.

**Study Selection**

Studies were considered eligible if they: (1) designed as randomized controlled trials (RCTs) or observational cohorts; (2) reported the impact of vitamin D and AF; (3) made available a quantitative measure of vitamin D and the number of AF cases in each vitamin D category for the dose-response analysis. For multiple publications/reports created from the same data, the studies with the longest follow-up period or the largest number of AF cases were included. In addition, certain publication types (e.g., reviews, editorials, letters, conference abstracts, and animal studies), or studies with insufficient data were excluded from this analysis.

**Data Extraction and Quality Assessment**

For each study, the basic characteristics were extracted, mainly including the first author, publication year, geographical location, study type, participants (sex, age, and sample size), duration of follow-up, adjustments for confounders, AF type (on-set or recurrence) methods of measuring vitamin D levels, season in which the study was conducted, categories of vitamin D and adjusted risk ratios (RRs) (categorical or continuous) with its 95% confidence intervals (CIs). If both unadjusted and adjusted RRs existed in one study, we extracted the most completely adjusted one.

We used the Newcastle-Ottawa quality assessment scale (NOS) to evaluate the quality for all included studies[3]. The validated NOS items with a total of 9 stars involved three aspects including the selection of population, the comparability of study, and the assessment of the outcome. In this meta-analysis, a NOS score of ≥6 stars was regarded as high-quality, otherwise, as low-quality studies[4].

**Patient involvement**

The ethical approval was not necessary because we performed this meta-analysis by collecting the data from published studies. No patients were involved in setting the research question, the outcome measures, the design, the implementation of the study, or the dissemination of the results.

**Statistical Analyses**

Both linear and nonlinear model was performed. We calculated study-specific RR (vitamin D per 10 ng/ml increase) and 95% CIs from the natural logs of the reported RRs and CIs across categories of vitamin D by using the method of Greenland and Longnecker[5]. Summary RRs and 95% CIs for a 10 unit increment in vitamin D were pooled using a random effects model. All data were analyzed by Review Manager version 5.30 software (the Nordic Cochrane Center, Rigshospitalet, Denmark) and Stata software (Version 14.0, Stata Corp LP, College Station, Texas, USA).

We performed the non-linear dose-response analysis by using the robust error meta-regression method (REMR) described by *Xu et al*.[6] This method is based on a “one-stage approach” which treating each study as a cluster of the whole sample and considering the within study correlations by clustered robust error. It requires known levels of vitamin D and RRs with variance estimates for at least two quantitative exposure categories. For studies that did not set the highest vitamin D group as a reference, data were transformed using a method described by *Hamling et al*.[7] which requires the number of cases and participants in each category (for detailed calculations, see Supplement File 2). If these data could not be obtained from an article, the evidence was not pooled. The category vitamin D concentration within each study for dose-response meta-analysis was calculated according to the methods of previous study. To assess the heterogeneity of RRs across studies, the I*^2^* (95% CI) statistic was calculated with the following interpretation: low heterogeneity, defined as I*^2^* < 50%; moderate heterogeneity, defined as I*^2^* 50% to 75%; and high heterogeneity, defined as I*^2^* >75%[8]. If there was evidence of publication bias, we additionally applied trim and fill methods to adjust for publication bias. Sensitivity analyses excluding one study at a time were conducted to clarify whether the results were simply due to one large study or a study with an extreme result. A P value < 0.05 was considered statistically significant.

1. Zhang Z, Yang Y, Ng CY, Wang D, Wang J, Li G, Liu T: **Meta-analysis of Vitamin D Deficiency and Risk of Atrial Fibrillation.** *Clin Cardiol* 2016, **39:**537-543.

2. Huang WL, Yang J, Yang J, Wang HB, Yang CJ, Yang Y: **Vitamin D and new-onset atrial fibrillation: A meta-analysis of randomized controlled trials.** *Hellenic J Cardiol* 2018, **59:**72-77.

3. Mcpheeters ML: **Newcastle-Ottawa Quality Assessment Scale.** 2012.

4. Aune D, Sen A, Norat T, Janszky I, Romundstad P, Tonstad S, Vatten LJ: **Body Mass Index, Abdominal Fatness, and Heart Failure Incidence and Mortality: A Systematic Review and Dose-Response Meta-Analysis of Prospective Studies.** *Circulation* 2016, **133:**639-649.

5. Greenland S, Longnecker MP: **Methods for trend estimation from summarized dose-response data, with applications to meta-analysis.** *American Journal of Epidemiology* 1992, **135:**1301-1309.

6. Xu C, Sar D: **The robust error meta-regression method for dose-response meta-analysis.** *International journal of evidence-based healthcare* 2017**:**1.

7. Hamling J, Lee P, Weitkunat R, Ambuhl M: **Facilitating meta-analyses by deriving relative effect and precision estimates for alternative comparisons from a set of estimates presented by exposure level or disease category.** *Stat Med* 2008, **27:**954-970.

8. Liu X, Ma J, Huang L, Zhu W, Yuan P, Wan R, Hong K: **Fluoroquinolones increase the risk of serious arrhythmias: A systematic review and meta-analysis.** *Medicine (Baltimore)* 2017, **96:**e8273.

**SUPPLEMENTAL TABLES**

| **Section/topic** | | **#** | | **Checklist item** | | **Reported on page #** | |
| --- | --- | --- | --- | --- | --- | --- | --- |
| **TITLE** | | | | | |  | |
| Title | | 1 | | Identify the report as a systematic review, meta-analysis, or both. | | 1 | |
| **ABSTRACT** | | | | | |  | |
| Structured summary | | 2 | | Provide a structured summary including, as applicable: background; objectives; data sources; study eligibility criteria, participants, and interventions; study appraisal and synthesis methods; results; limitations; conclusions and implications of key findings; systematic review registration number. | | 1 | |
| **INTRODUCTION** | | | | | |  | |
| Rationale | | 3 | | Describe the rationale for the review in the context of what is already known. | | 3 | |
| Objectives | | 4 | | Provide an explicit statement of questions being addressed with reference to participants, interventions, comparisons, outcomes, and study design (PICOS). | | 3-4 | |
| **METHODS** | | | | | |  | |
| Protocol and registration | | 5 | | Indicate if a review protocol exists, if and where it can be accessed (e.g., Web address), and, if available, provide registration information including registration number. | | 4 | |
| Eligibility criteria | | 6 | | Specify study characteristics (e.g., PICOS, length of follow-up) and report characteristics (e.g., years considered, language, publication status) used as criteria for eligibility, giving rationale. | | 4 | |
| Information sources | | 7 | | Describe all information sources (e.g., databases with dates of coverage, contact with study authors to identify additional studies) in the search and date last searched. | | 4 | |
| Search | | 8 | | Present full electronic search strategy for at least one database, including any limits used, such that it could be repeated. | | 4 | |
| Study selection | | 9 | | State the process for selecting studies (i.e., screening, eligibility, included in systematic review, and, if applicable, included in the meta-analysis). | | 4 | |
| Data collection process | | 10 | | Describe method of data extraction from reports (e.g., piloted forms, independently, in duplicate) and any processes for obtaining and confirming data from investigators. | | 5 | |
| Data items | | 11 | | List and define all variables for which data were sought (e.g., PICOS, funding sources) and any assumptions and simplifications made. | | 5 | |
| Risk of bias in individual studies | | 12 | | Describe methods used for assessing risk of bias of individual studies (including specification of whether this was done at the study or outcome level), and how this information is to be used in any data synthesis. | | 5 | |
| Summary measures | | 13 | | State the principal summary measures (e.g., risk ratio, difference in means). | | 5 | |
| Synthesis of results | | 14 | | Describe the methods of handling data and combining results of studies, if done, including measures of consistency (e.g., I^2^) for each meta-analysis. | | 5 | |
| Section/topic | | # | | Checklist item | | Reported on page # | |
| Risk of bias across studies | | 15 | | Specify any assessment of risk of bias that may affect the cumulative evidence (e.g., publication bias, selective reporting within studies). | | 5 | |
| Additional analyses | | 16 | | Describe methods of additional analyses (e.g., sensitivity or subgroup analyses, meta-regression), if done, indicating which were pre-specified. | | 5 | |
| **RESULTS** | | | | | |  | |
| Study selection | | 17 | | Give numbers of studies screened, assessed for eligibility, and included in the review, with reasons for exclusions at each stage, ideally with a flow diagram. | | 5 | |
| Study characteristics | | 18 | | For each study, present characteristics for which data were extracted (e.g., study size, PICOS, follow-up period) and provide the citations. | | 5 | |
| Risk of bias within studies | | 19 | | Present data on risk of bias of each study and, if available, any outcome level assessment (see item 12). | | 6 | |
| Results of individual studies | | 20 | | For all outcomes considered (benefits or harms), present, for each study: (a) simple summary data for each intervention group (b) effect estimates and confidence intervals, ideally with a forest plot. | | 6 | |
| Synthesis of results | | 21 | | Present results of each meta-analysis done, including confidence intervals and measures of consistency. | | 6-7 | |
| Risk of bias across studies | | 22 | | Present results of any assessment of risk of bias across studies (see Item 15). | | 7-8 | |
| Additional analysis | | 23 | | Give results of additional analyses, if done (e.g., sensitivity or subgroup analyses, meta-regression [see Item 16]). | | 7 | |
| **DISCUSSION** | | | | | |  | |
| Summary of evidence | | 24 | | Summarize the main findings including the strength of evidence for each main outcome; consider their relevance to key groups (e.g., healthcare providers, users, and policy makers). | | 8-11 | |
| Limitations | | 25 | | Discuss limitations at study and outcome level (e.g., risk of bias), and at review-level (e.g., incomplete retrieval of identified research, reporting bias). | | 11 | |
| Conclusions | | 26 | | Provide a general interpretation of the results in the context of other evidence, and implications for future research. | | 12 | |
| **FUNDING** | | | | | |  | |
| Funding | | 27 | | Describe sources of funding for the systematic review and other support (e.g., supply of data); role of funders for the systematic review. | | 12 | |

**Table S2: Search strategy**
**PubMed database**

| Search | Query | Results |
| --- | --- | --- |
| #1 | heart rate | 226524 |
| #2 | pulse rate | 6707 |
| #3 | atrial fibrillation | 72822 |
| #4 | atrial flutter | 7928 |
| #5 | atrial tachycardia | 3258 |
| #6 | supraventricular tachycardia | 5847 |
| #7 | vitamin D | 64280 |
| #8 | 25(OH)D | 492 |
| #9 | #1 OR #2 OR #3 OR #4 OR #5 OR #6 | 306963 |
| #10 | #7 OR #8 | 64294 |
| #11 | #9 AND #10 | 176 |

**Embase database**

| Search | Query | Results |
| --- | --- | --- |
| #1 | heart rate | 294626 |
| #2 | pulse rate | 56108 |
| #3 | atrial fibrillation | 150434 |
| #4 | heart atrium flutter | 13299 |
| #5 | atrial tachycardia | 20166 |
| #6 | supraventricular tachycardia | 23282 |
| #7 | vitamin D | 154056 |
| #8 | 25(OH)D | 13858 |
| #9 | #1 OR #2#3 OR #4 OR #5 OR #6 | 502788 |
| #10 | #7 OR #8 | 154410 |
| #11 | #9 AND #10 | 1205 |

**Cochrane library**

| Search | Query | Results |
| --- | --- | --- |
| #1 | heart rate | 44042 |
| #2 | pulse rate | 3450 |
| #3 | atrial fibrillation | 9282 |
| #4 | atrial flutter | 653 |
| #5 | atrial tachycardia | 210 |
| #6 | supraventricular tachycardia | 674 |
| #7 | vitamin D | 8622 |
| #8 | 25(OH)D | 1727 |
| #9 | #1 OR #2 OR #3 OR #4 OR #5 OR #6 | 55579 |
| #10 | #7 OR #8 | 8677 |
| #11 | #9 AND #10 | 103 |


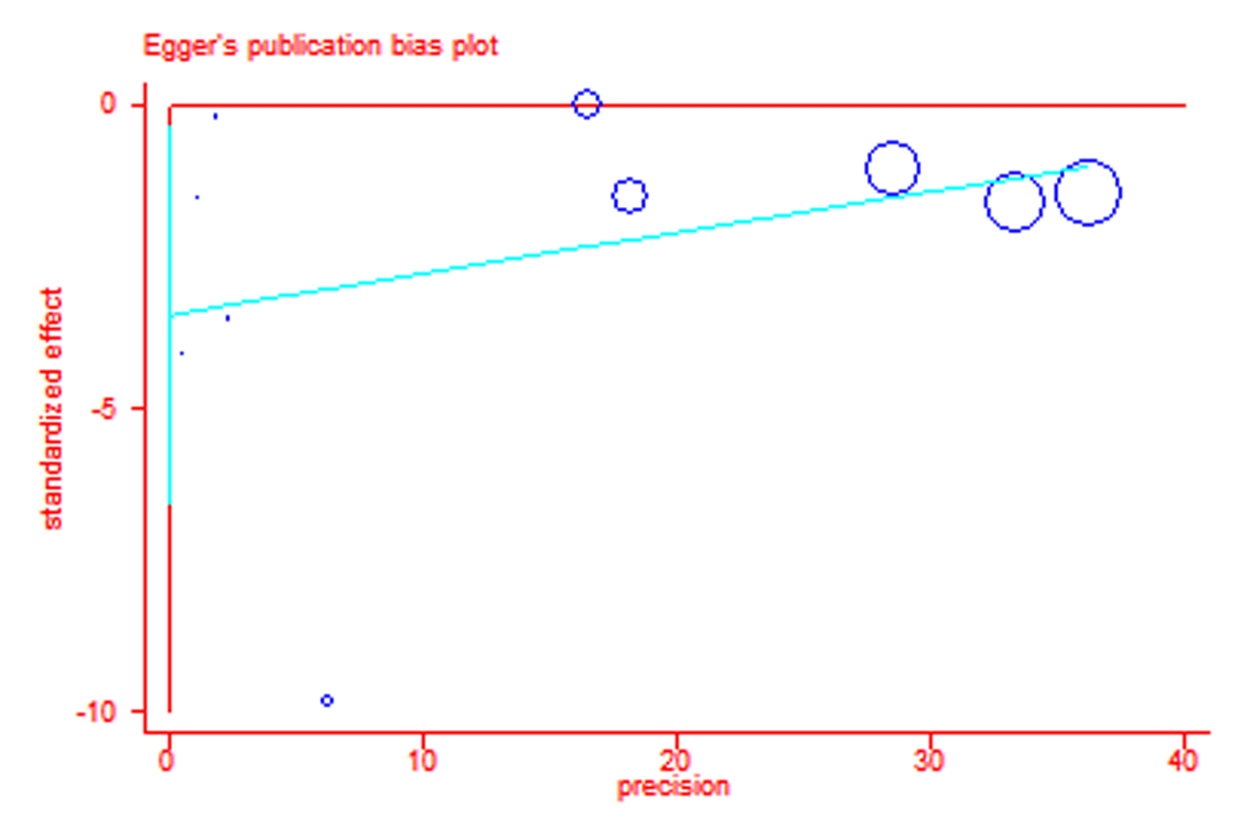


**Figure S1. Eegg’s test of Publication bias for the association between vitamin D and AF.**

Abbreviations: AF = atrial fibrillation; SE = standard error


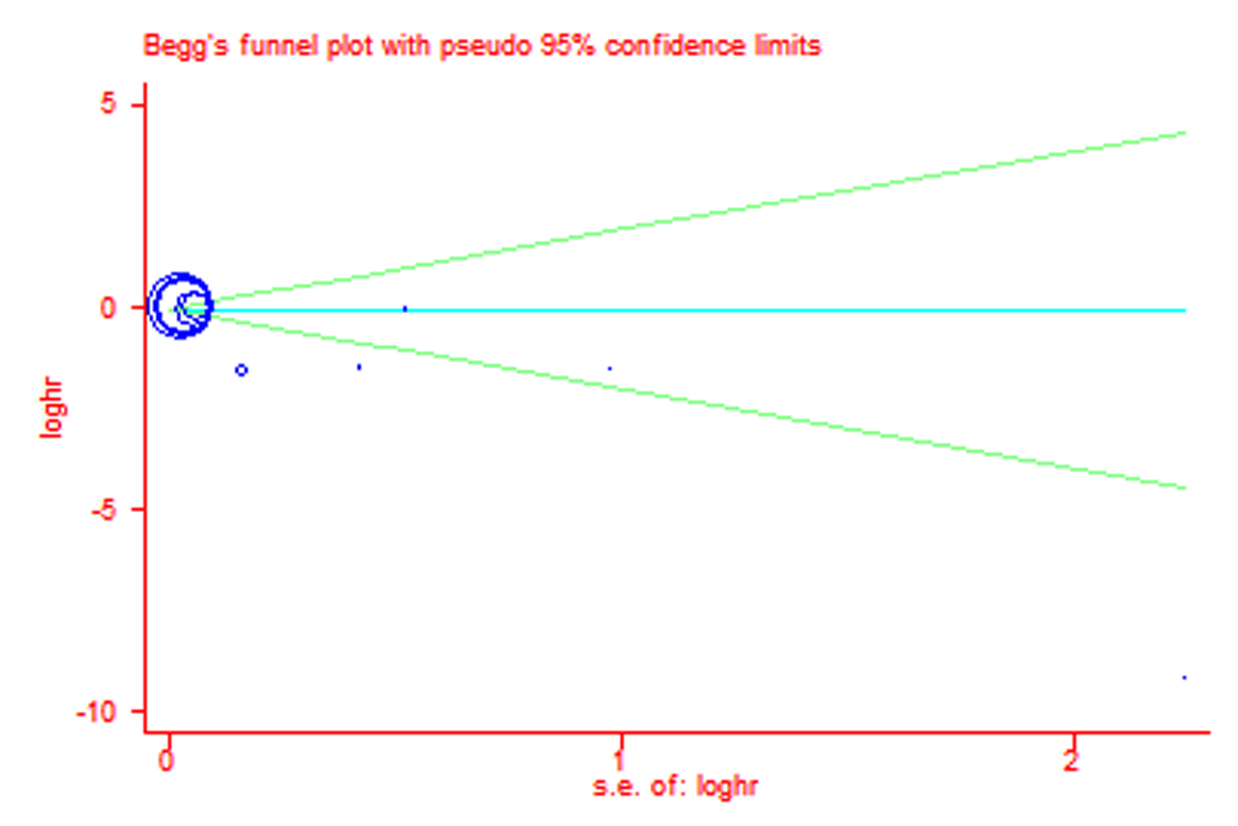


**Figure S2.Begg’s test of Publication bias for the association between between vitamin D and AF**

Abbreviations: AF = atrial fibrillation; SE = standard error


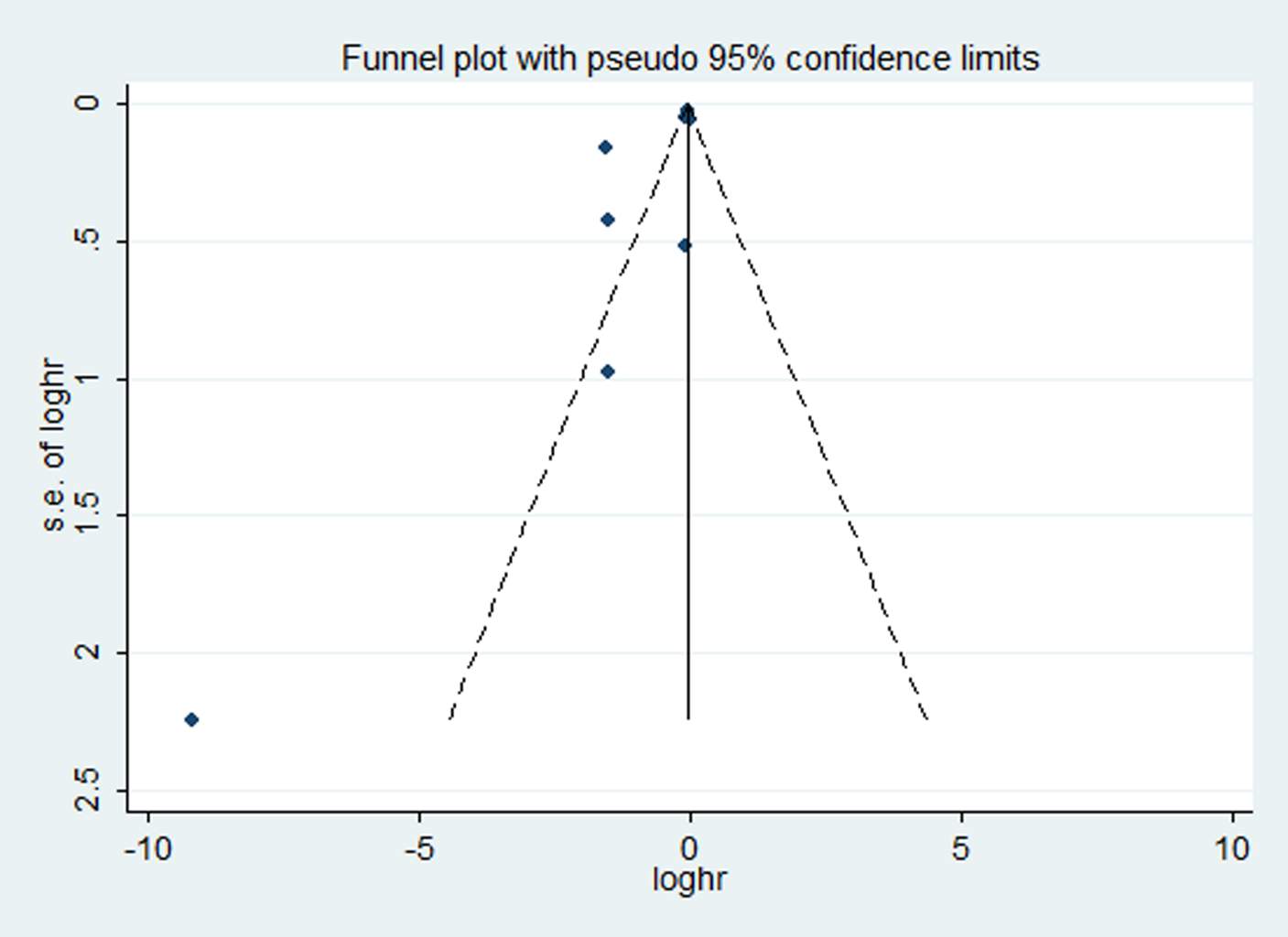


**Figure S3.Funnel Plot of Publication bias for the association between between vitamin D and AF**

Abbreviations: AF = atrial fibrillation; SE = standard error
